# Moderate (2%, *v*/*v*) Ethanol Feeding Alters Hepatic Wound Healing after Acute Carbon Tetrachloride Exposure in Mice

**DOI:** 10.3390/biom6010005

**Published:** 2016-01-06

**Authors:** Krutika T. Deshpande, Shinlan Liu, Jennifer M. McCracken, Lu Jiang, Ta Ehpaw Gaw, Lindsey N. Kaydo, Zachary C. Richard, Maura F. O’Neil, Michele T. Pritchard

**Affiliations:** 1 Department of Pharmacology, Toxicology and Therapeutics, University of Kansas Medical Center, 3901 Rainbow Blvd, Kansas City, KS 66160, USA; kdeshpande@outlook.com (K.T.D.); jawahal@hotmail.com (S.L.); jmccracken2@kumc.edu (J.M.M.); ljiang@kumc.edu (L.J.); tagaw75@gmail.com (T.E.G.); moneil@kumc.edu (M.F.O.); 2 Department of Pathology, University of Kansas Medical Center, 3901 Rainbow Blvd, Kansas City, KS 66160, USA; 3 Department of Gastroenterology and Hepatology, Case Western Reserve University, 10900 Euclid Ave., Cleveland, OH 44106, USA; lxk176@case.edu (L.N.K.); zrichar1@nd.edu (Z.C.R.)

**Keywords:** ethanol, carbon tetrachloride, inflammation, liver regeneration, matrix remodeling, wound healing, fibrosis

## Abstract

Wound healing consists of three overlapping phases: inflammation, proliferation, and matrix synthesis and remodeling. Prolonged alcohol abuse can cause liver fibrosis due to deregulated matrix remodeling. Previous studies demonstrated that moderate ethanol feeding enhances liver fibrogenic markers and frank fibrosis independent of differences in CCl_4_-induced liver injury. Our objective was to determine whether or not other phases of the hepatic wound healing response were affected by moderate ethanol after CCl_4_ exposure. Mice were fed moderate ethanol (2% *v*/*v*) for two days and then were exposed to CCl_4_ and euthanized 24–96 h later. Liver injury was not different between pair- and ethanol-fed mice; however, removal of necrotic tissue was delayed after CCl_4_-induced liver injury in ethanol-fed mice. Inflammation, measured by TNFα mRNA and protein and hepatic *Ly6c* transcript accumulation, was reduced and associated with enhanced hepatocyte apoptosis after ethanol feeding. Hepatocytes entered the cell cycle equivalently in pair- and ethanol-fed mice after CCl_4_ exposure, but hepatocyte proliferation was prolonged in livers from ethanol-fed mice. CCl_4_-induced hepatic stellate cell activation was increased and matrix remodeling was prolonged in ethanol-fed mice compared to controls. Taken together, moderate ethanol affected each phase of the wound healing response to CCl_4_. These data highlight previously unknown effects of moderate ethanol exposure on hepatic wound healing after acute hepatotoxicant exposure.

## 1. Introduction

Ethanol consumption contributes to liver injury and subsequent alterations in hepatic structure and function. If prolonged, ethanol exposure can precipitate the progression of liver disease from simple steatosis to more severe liver disease states. Indeed, in a certain percentage of chronic alcohol abusers, an unrelenting liver wound healing response promotes the development of fibrosis, cirrhosis and even hepatocellular carcinoma [1,2,3]. While our understanding of how derangements in hepatic wound healing contribute to fibrosis have grown considerably over the past decades, these discoveries have not lead to improved therapies. Indeed, liver transplant remains the only way to cure advanced liver disease. Due to increasing demand for transplantable livers and a dwindling supply of suitable organs, additional studies are required to improve therapeutic options for advanced liver disease patients. Certainly, a more complete understanding of the specific contributions ethanol makes to liver injury and subsequent hepatic wound healing would be beneficial.

A cascade of sequential and overlapping steps are involved in wound healing whose outcome is to reinstate tissue integrity and function [4]. These steps are often categorized into three phases: the inflammatory, proliferative and remodeling phases. The inflammatory phase includes hemostasis, as well as activation of various humoral and cellular innate immune system components. Inflammation is responsible for debriding the wound of pathogens and cell debris, and for providing some of the signals required to induce the proliferative phase. The proliferative phase includes production of new epithelial and endothelial cells and tissue-resident fibroblasts. Activated fibroblasts contribute to the synthesis of a provisional ECM over which new epithelial and endothelial cells migrate to areas in need of repair. ECM remodeling is the last phase of wound healing. Early in this phase, additional ECM is synthesized in which type I collagen is a predominant component, while later in the process, ECM degradation occurs during which the once injured tissue is reorganized closely approximating its original structural and functional properties. Perturbations in this process result in wound healing abnormalities. For example, wounds in diabetic patients do not heal due to, in part, persistent inflammation leading to ulcers [5]. Likewise, overly robust wound healing can precipitate development of scars or solid organ fibrosis [4]. While best described for skin wounds, this sequence of steps occurs in other organs, including the liver, but the precise mechanisms responsible for solid organ wound repair are incompletely understood.

Several animal models are employed to mimic different drinking patterns, and resultant liver injury, in alcohol abusers [6]. While informative regarding ethanol’s role in development of steatosis and limited injury, inflammation, and liver regeneration, these models do not adequately represent long-term, iterative liver injury and incomplete hepatic wound healing, which drives fibrogenesis. Recently, two new animal models were developed which combine carbon tetrachloride (CCl_4_), a model hepatotoxicant, with ethanol exposure. In one of those models, mice are fed a high-dose ethanol-containing diet (5% *v*/*v*, 35% total caloric intake) [7] while in the other, mice are fed a moderate-dose ethanol-containing diet (2% *v*/*v*, 11% total caloric intake) [8,9]. In the former, cytochrome P450 2E1 (CYP2E1), an enzyme required for CCl_4_’s bioactivation and hepatotoxicity, is induced in response to chronic ethanol exposure, while in the latter, CYP2E1 is not induced. In both models, mice are exposed to CCl_4_, but the dose is reduced in mice fed 5% ethanol-containing diets to ensure equivalent liver injury between diet groups. After 2% ethanol feeding, liver injury is equivalent in pair- and ethanol-fed mice without a need to adjust the CCl_4_ dose.

Published studies utilizing these two animal models demonstrate that moderate ethanol accelerates fibrogenesis and fibrosis after acute and chronic CCl_4_ exposure, respectively. Natural killer cell inhibition [7], increased adenosine receptor signaling [8] and hepatocyte apoptosis [10] all contribute to enhanced hepatic stellate cell (HSC) activation and fibrosis in ethanol-fed mice. Aside from ethanol’s effects on HSC activation and fibrosis, other phases of the hepatic wound healing response which likely contribute to enhanced fibrosis after have not been explored in detail.

The objective of this study was to evaluate various aspects of the hepatic wound healing response after acute CCl_4_ in mice fed moderate ethanol-containing diets. Specifically, we wished to determine whether or not inflammation, proliferation and matrix remodeling were altered by moderate ethanol after CCl_4_ exposure. Due to the fact that inflammation contributes to hepatoprotection [11,12,13] and liver regeneration [14,15,16,17], we tested the hypothesis that moderate ethanol feeding reduced inflammation leading to increased hepatocyte apoptosis observed by others [10]. Moreover, we hypothesized that reduced inflammation would inhibit liver regeneration, further promoting the pro-fibrogenic milieu in the injured liver as shown previously [10]. When taken together, we predicted that moderate ethanol perturbed every phase of the hepatic wound healing response.

## 2. Results and Discussion

### 2.1. The Effects of Moderate (2% v/v) Ethanol on Hepatic Cytochrome P450 2E1 (CYP2E1), Hepatic Injury, Steatosis and Removal of Necrotic Tissue after Acute Carbon Tetrachloride (CCl_4_) Exposure

CCl_4_ is a hepatotoxicant widely used to model human acute and chronic liver injury in mice and rats. CCl_4_ requires metabolism (bioactivation), *in vivo*, by cytochrome P450 2E1 (CYP2E1) for its hepatotoxicity [18]. CYP2E1-induced bioactivation of CCl_4_ leads to the production of highly reactive metabolites including the trichloromethyl (CCl_3_*) and trichloromethylperoxy (CCl_3_OO*) radicals [19]. In the liver, the highest concentration of CYP2E1 is in the pericentral area (Zone 3) [20], which is where liver injury is localized after CCl_4_ exposure. After CYP2E1-mediated bioactivation, these reactive metabolites modify proteins, nucleic acids and lipids. In particular, hepatocyte membrane lipid peroxidation leads to necrotic cell death [19]. Liver injury can expand from the site of CCl_4_ bioactivation by a “bystander effect”, leading to additional necrotic and apoptotic cell death [21,22,23].

**Figure 1:**
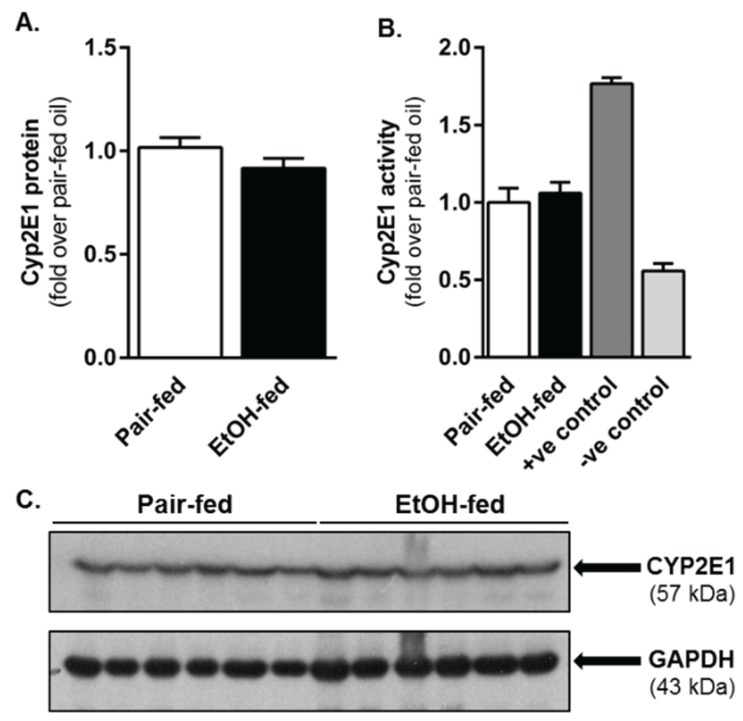
Hepatic CYP2E1 content and activity did not differ between control (pair-fed) or ethanol-fed mice. Mice were allowed free-access to a 2% (*v*/*v*) ethanol-containing Lieber-DiCarli diet, or pair-fed a control diet, for 10 days. Livers were harvested, a portion of which was used for performing a CYP2E1 immunoblot (**A**,**C**) while a separate portion was used to perform a CYP2E1 activity assay (**B**). In (**B**), the positive (+ve) control was generated by using microsomes isolated from mice fed a 5% (*v*/*v*) ethanol-containing diet for four weeks. The negative (−ve) control was generated by using microsomes isolated from mice 24 h after a single CCl_4_ exposure (CCl_4_ consumptively depletes CYP2E1). *N* = 4–8 mice per group.

Ethanol is a known inducer of cytochrome P450 2E1 (CYP2E1) [24,25] and could increase CCl_4_’s hepatotoxicity in ethanol-fed mice. Therefore, we evaluated hepatic CYP2E1 content and activity. Two percent (2%, *v*/*v*) moderate ethanol exposure did not increase the hepatic content of CYP2E1 or CYP2E1 activity above that found in pair-fed mice (Figure 1).

**Figure 2:**
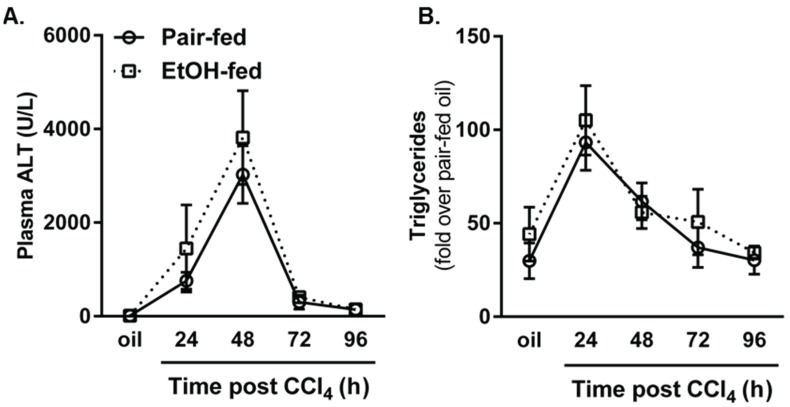
CCl_4_-induced liver injury and steatosis were not affected by ethanol feeding to mice. Mice were allowed free-access to a 2% (*v*/*v*) ethanol containing diet for two days and then were exposed to CCl_4_ and euthanized 24, 48, 72 or 96 h thereafter, while remaining on the ethanol diet. Control animals were pair-fed a diet that isocalorically substituted maltose dextrins for ethanol. (**A**) Plasma ALT was used to determine hepatic injury; (**B**) A biochemical assay was used to quantify hepatic triglyceride content. *N* = 4–8 mice per group.

CCl_4_ induced robust liver injury, as determined by measuring plasma alanine aminotransferase (ALT) activities, which was greatest 48 h after hepatotoxicant exposure and subsided thereafter; importantly, there was no difference in plasma ALT activities between groups (Figure 2A). These data parallel those found by others using the same ethanol-feeding paradigm [8,10]. Likewise, hepatic steatosis, as determined by a biochemical assay for triglyceride, increased 24 h after CCl_4_ and was reduced thereafter without a difference between pair- or ethanol-fed groups at any time point (Figure 2B). Taken together, these data suggest that moderate ethanol does not alter CCl_4_-induced liver injury or steatosis. Thus, this model provides a way to evaluate the impact of ethanol on hepatic wound healing independent of differences in the severity of CCl_4_-induced, necrotic liver injury.

**Figure 3:**
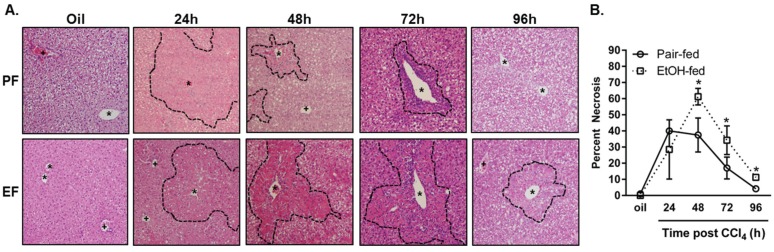
Moderate ethanol feeding delayed removal of nectrotic tissue after CCl_4_ exposure. Mice were allowed free-access to a 2% (*v*/*v*) ethanol containing diet for 2 days and then were exposed to CCl_4_ and euthanized 24, 48, 72 or 96 h thereafter while remaining on the ethanol diet. Control animals were pair-fed a diet that isocalorically substituted maltose dextrins for ethanol. (**A**) Representative micrographs taken of hematoxylin and eosin stained liver sections taken at the time points indicated. The dashed line demarks boundary of injured (24 h) or necrotic hepatocytes (48, 72, 96 h). PF = pair-fed, EF = ethanol-fed, asterisk = central vein, plus sign = portal vein; (**B**) Percent necrotic area graphed over time post CCl_4_ in pair- and ethanol-fed mice. *N* = 4–8 mice per group.

ALT has a half-life of about 3 days [26]. Although ALT is an appropriate measure of hepatocyte injury and necrosis, it does not indicate whether or not differences exist in the removal of the necrotic tissue from which ALT was liberated after CCl_4_ exposure. To this end, histopathological analysis was employed to determine whether or not ethanol feeding affected the ability to remove necrotic tissue. This analysis revealed an increased percentage of necrotic tissue in livers from ethanol-fed mice 48, 72 and 96 h after CCl_4_ exposure relative to necrosis in pair-fed mice at the same time points (Figure 3). Liver to body weight ratios paralleled this increase in necrotic tissue area in ethanol-fed mice.

Because liver injury was not different between diet groups, these data suggest that there is an impaired ability of the ethanol-exposed liver to remove necrotic tissue (Table 1).

**Table 1:** Initial and final body weights, liver weights and liver to body weight ratios.

	PAIR-FED				EtOH-FED			
Exp. Group	Initial BW (g)	Final BW (g)	Liver Weight (g)	Liver to Body Weight Ratio (%)	Initial BW (g)	Final BW (g)	Liver Weight (g)	Liver to Body Weight Ratio (%)
Oil	20.0 (0.9)	21.2 (1.0)	0.971 (0.052)	4.6 (0.250)	19.1 (0.4)	20.9 (0.6)	0.949 (0.066)	4.5 (0.214)
24 h	18.8 (0.5)	21.1 (1.1)	0.978 (0.056)	4.6 (0.100)	17.3 (0.7)	18.9 (0.6)	0.964 (0.054)	5.1 (0.212) *
48 h	17.7 (0.7)	19.4 (0.8)	0.936 (0.031)	4.7 (0.048)	18.8 (0.9)	19.6 (1.0)	0.988 (0.029)	5.1 (0.175) *
72 h	19.7 (0.6)	20.7 (0.9)	1.001 (0.068)	4.8 (0.151)	19.8 (0.5)	20.7 (0.8)	1.081 (0.028)	5.3 (0.132) *
96 h	21.4 (0.3)	21.2 (0.7)	1.295 (0.038)	4.9 (0.155)	21.0 (0.2)	20.9 (0.2)	1.115 (0.076)	5.3 (0.312)

Standard error of the mean, in parentheses, is found below the mean value for each group. * *p* < 0.05 relative to pair-fed.

### 2.2. Markers of Inflammation and Hepatocyte Apoptosis after Acute CCl_4_ Exposure: Modulation by Moderate Ethanol

#### 2.2.1. TNFα Production and Hepatic Macrophages

**Figure 4:**
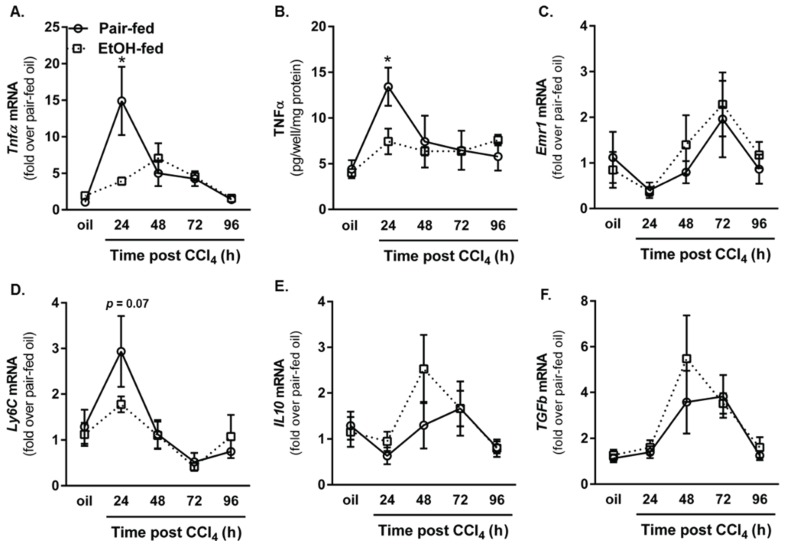
Ethanol feeding suppressed hepatic inflammation early after CCl_4_ exposure. Real-time PCR (**A**) was used to determine hepatic *Tnfα* transcript level, while an ELISA (**B**) was used to determine TNFα concentration in peripheral blood from pair- and ethanol-fed mice after CCl_4_. Hepatic transcripts for the F4/80 gene (*Emr1* (**C**); *Ly6C* (**D**); *Il10* (**E**) and *Tgfβ* **(F**)) were determined using real-time PCR. For real time PCR, data are expressed as fold change over pair-fed, olive oil exposed mice after normalization to 18S. *N* = 4–8 mice per group. * *p* <0.05.

Inflammation is one of the first responses to tissue injury [27]. The innate immune system, including humoral (complement activation) and cellular (macrophages and neutrophils) components, induces a rapid inflammatory response to invading organisms and/or tissue debris. Tumor necrosis factor (TNF)α is a proinflammatory molecule predominantly produced by activated macrophages [28]. TNFα is also important for hepatoprotection [11,12,13,29] and liver regeneration [14,15,16,17]. As a surrogate marker for inflammation, we measured hepatic accumulation of TNFα mRNA as well as plasma TNFα protein levels in pair- and ethanol-fed mice. In contrast to published literature that shows TNFα is increased after exposure to 2% ethanol for two days or after chronic ethanol feeding (5% for four weeks) [30], CCl_4_-induced TNFα was suppressed by moderate ethanol feeding to mice, 24 h after CCl_4_ exposure (total of 4 d on 2% ethanol, Figure 4A,B).

Because macrophages are major producers of TNFα, we measured hepatic accumulation of *Emr1* (gene encoding F4/80, a mouse macrophage marker [31]) and *Ly6C*, a marker associated with inflammatory/M1 macrophages recruited to the liver after injury [31,32]. While *Emr1* transcripts were reduced 24 h after CCl_4_ exposure in both groups of mice, ethanol-feeding did not affect the level of this transcript (Figure 4C). By contrast, CCl_4_ increased hepatic accumulation of *Ly6C* transcripts in pair-fed mice, but not ethanol-fed mice, 24 h after CCl_4_ exposure; this approached statistical significance (Figure 4D, *p* = 0.07). These data suggested that moderate ethanol exposure may have shifted hepatic macrophage populations towards a wound healing/M2-like phenotype which could promote fibrogenesis [33,34]. To address this point, we measured accumulation of hepatic *Il10* and *Tgfβ* transcripts. Moderate ethanol feeding did not alter hepatic *Il10* or *Tgfβ* (Figure 4E,F). Future work is needed to delineate which macrophage subset is required for TNFα production in response to CCl_4_. Specifically, it is important to determine whether or not resident macrophages change their phenotype or if early macrophage recruitment is required for robust TNFα production after CCl_4_ in pair-fed mice. Analysis of other inflammatory cytokines or chemokines may provide additional insight on how moderate ethanol alters the hepatic microenvironment to shape wound healing after acute CCl_4_ exposure.

##### 2.2.2. Hepatocyte Apoptosis

**Figure 5:**
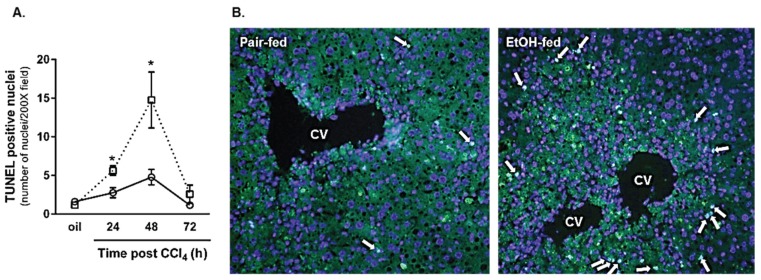
Hepatocyte apoptosis was increased in livers from ethanol-fed mice 24 and 48 h after CCl_4_ exposure. The terminal deoxynucleotidyl transferase dUTP nick end labeling (TUNEL) assay was used to determine the number of apoptotic hepatocyte nuclei in livers from pair- and ethanol-fed mice 24, 48, and 72 h after CCl_4_ exposure. (**A**) Quantification of the number of TUNEL-positive hepatocyte nuclei in each 200× image (three non-overlapping images per animal); (**B**) Representative TUNEL staining from pair- and ethanol-fed mice 48 h (maximum hepatocyte apoptosis) after CCl_4_ exposure. *N* = 4 mice per group. * *p* < 0.05.

CCl_4_ causes predominantly necrotic liver injury [19] but hepatocyte apoptosis also occurs and contributes to hepatocyte loss [23]. Apoptosis was observed in livers from both diet groups after CCl_4_ exposure (Figure 5). However, consistent with impaired hepatoprotection found in livers with reduced TNFα [13], hepatocyte apoptosis was further increased in livers from ethanol-fed mice 24 and 48 h after CCl_4_ (Figure 5). The apoptosis occurred outside the area of hepatocyte necrosis caused by CCl_4_. These data are consistent with the work of others [10] and suggest that hepatocyte survival and/or sensitivity to apoptosis-inducing signals was impaired in livers from ethanol-fed mice. Taken together, moderate ethanol suppressed hepatic TNFα production, which may be related to differences in macrophage populations recruited to the liver after acute CCl_4_ exposure, and was associated with increased hepatocyte apoptosis.

### 2.3. Impact of Moderate Ethanol Feeding to Mice on Liver Regeneration after Acute CCl_4_

#### 2.3.1. Dynamics of Hepatic Cyclin Content

**Figure 6:**
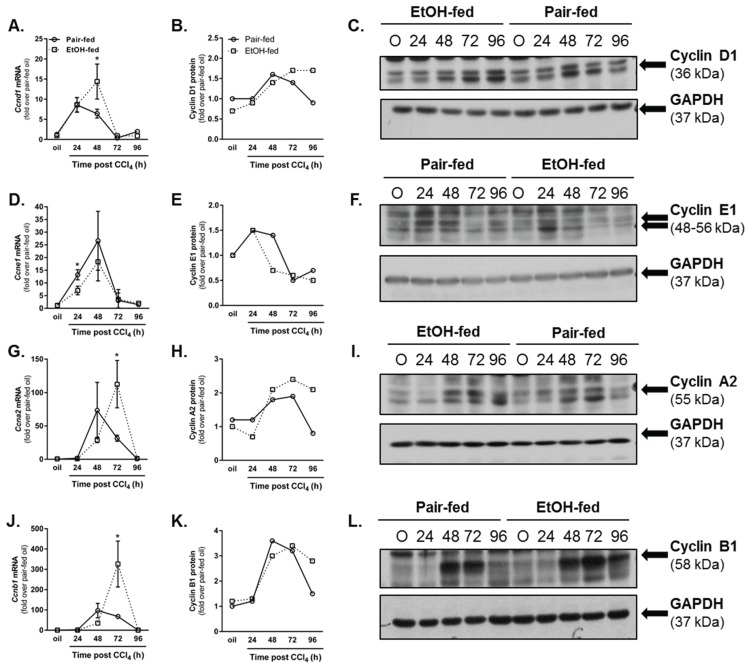
Moderate ethanol feeding enhanced hepatic cyclin content after CCl_4_ exposure. Cyclin D1 (**A**–**C**), cyclin E1 (**D**–**F**), cyclin A2 (**G**–**I**) and cyclin B1 (**J**–**L**) were each analyzed in livers from pair-and ethanol-fed mice after CCl_4_ exposure. In (**A**,**D**,**G**,**J**), real time PCR was used to determine hepatic content of cyclin transcripts while in (**C**,**F**,**I**,**L**), immunoblotting was used to determine hepatic cyclin protein content from pooled liver samples (n = 4–8 mice per group). Real time PCR data are expressed as fold change over pair-fed, olive oil exposed mice after normalization to 18S. Protein band integrated density was determined for each cyclin and GAPDH (loading control) band. After normalization to GAPDH, band densities were used to calculate fold change in cyclin protein over pair-fed mice exposed to olive oil (oil). *N* = 4–8 mice per group. * *p* < 0.05.

Inflammation after liver injury is required for timely liver regeneration [29,35]. Gut-derived bacterial cell wall components such as lipopolysaccharide, complement activation products and their cognate receptors, TNFα and IL6 are all implicated in the orchestration of timely liver regeneration [29,36,37,38]. Given that TNFα was reduced and hepatocyte cell death increased after CCl_4_-induced liver injury, we hypothesized that liver regeneration would also be impaired by ethanol feeding. To test this hypothesis, we evaluated the hepatic cyclin D1, E1, A2 and B1 transcript and protein accumulation. Cyclin D1 is the first cyclin transcript synthesized once hepatocytes enter the cell cycle [39]. Cyclin D1 mRNA increases equivalently in livers from pair- and ethanol-fed mice 24 h after CCl_4_ (Figure 6A). However, 48 h after CCl_4_, cyclin D1 transcript levels are reduced in pair-fed mice but maintained in ethanol-fed mice (Figure 6A). At the protein level, cyclin D1 content was sustained in livers from ethanol-fed mice relative to pair-fed mice 96 h after CCl_4_ exposure (Figure 6B,C). Few differences in cyclin E1, the cyclin whose expression peaks between the G1 and S phases of the cell cycle [40], were observed between pair- and ethanol-fed groups; only a small but significant reduction in cyclin E1 mRNA was observed in livers from ethanol-fed mice 24 h after CCl_4_ (Figure 6D). Hepatic cyclin E1 protein was reduced by 50% 48 h after CCl_4_ exposure in ethanol-fed mice, consistent with reduced cyclin E1 mRNA (Figure 6E,F).

Cyclin A2 peaks during the G2 phase of the cell cycle and is important for transition to mitosis [41,42]. Hepatic cyclin A2 transcript accumulation occurs in pair- and ethanol-fed mice. However, in contrast to pair-fed mice, ethanol-fed mice exhibited 3.5-fold higher cyclin A2 transcripts 72 h after CCl_4_ exposure (Figure 6G). Consistently, Cyclin A2 protein levels were more than twofold greater in livers from ethanol-fed mice 96 h after CCl_4_ exposure (Figure 6H,I). Cyclin B1 peaks at the G2-M phase transition [43]. At the mRNA level, cyclin B1 peaks in livers from pair-fed mice 48 h after CCl_4_, but this peak is delayed until 72 h after CCl_4_ and greater in livers from ethanol-fed mice (Figure 6J). At the protein level, the increase in cyclin B1 mRNA manifested in a 2.5-fold increase in cyclin B1 protein at 96 h after CCl_4_ (Figure 6K,L). Taken together, the cyclin data in Figure 6 suggest that moderate ethanol feeding did not impair hepatocyte entry into the cell cycle, but prolonged the cell cycle in liver after acute CCl_4_ exposure.

#### 2.3.2. Retinoblastoma Phosphorylation

**Figure 7:**
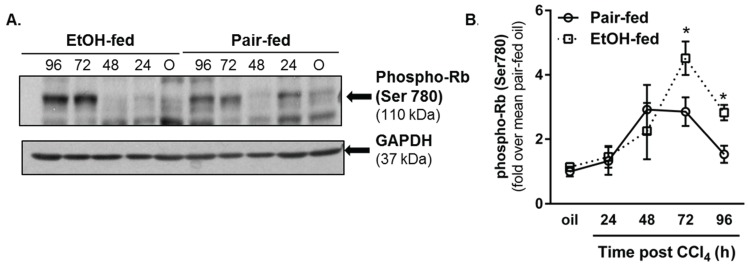
Phosphorylation of the retinoblastoma (Rb) protein was increased by moderate ethanol feeding to mice. Mice on ethanol-containing diets were exposed to CCl_4_ and euthanized 24–96 h later. Immunoblots were used to determine hepatic content of phospho-Rb (Ser780) in livers from each mouse. (**A**) Representative phospho-Rb immunoblot. GAPDH was used as a loading control; (**B**) Semi-quantification of band densities from all blots after normalization to GAPDH. Data are graphed as fold change over pair-fed, olive oil-treated mice. *N* = 4–8 mice per group. * *p* < 0.05.

Given the changes in hepatic cyclin expression, we asked if other liver regeneration markers were also affected after CCl_4_ exposure. To this end, we evaluated phosphorylation of the retinoblastoma (Rb) protein. When hypophosphorylated, Rb binds to the E2F transcription factor and prevents its induction of cell cycle regulators such as the cyclin genes. By contrast, Rb hyperphosphorylation inhibits Rb-mediated E2F sequestration, facilitating cyclin gene expression and cell cycle progression [44]. By immunoblotting, we found Rb phosphorylation was greater in livers from ethanol-fed mice compared to pair-fed mice 72 and 96 h after CCl_4_ exposure (Figure 7A,B). These data are consistent with increased cyclin D1, A2 and B1 content in livers from ethanol-fed mice 96 h after CCl_4_.

#### 2.3.3. Ki67 Staining and Mitotic Index

**Figure 8:**
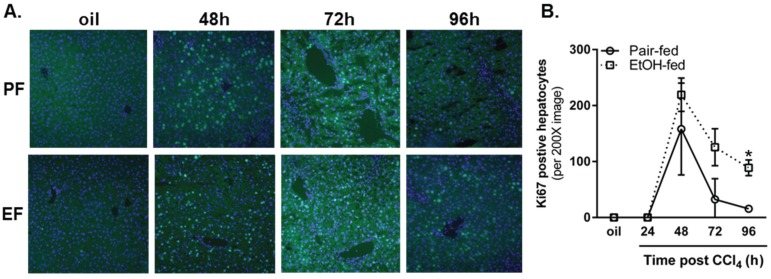
Moderate ethanol feeding to mice sustained hepatocyte proliferation late in the time course after CCl_4_ exposure. (**A**) Representative images from Ki67-stained livers from pair- and ethanol-fed mice by immunofluorescense (green) after CCl_4_. DAPI (blue) was used as a nuclear counterstain. Sections were stained on the same day and exposure times kept constant for each image; (**B**) Quantification of the number of Ki67-positive hepatocyte nuclei in 3–4 images from a single liver section per mouse. PF = Pair-fed, EF = Ethanol-fed. *N* = 3–4 mice per group. * *p* < 0.05.

**Figure 9:**
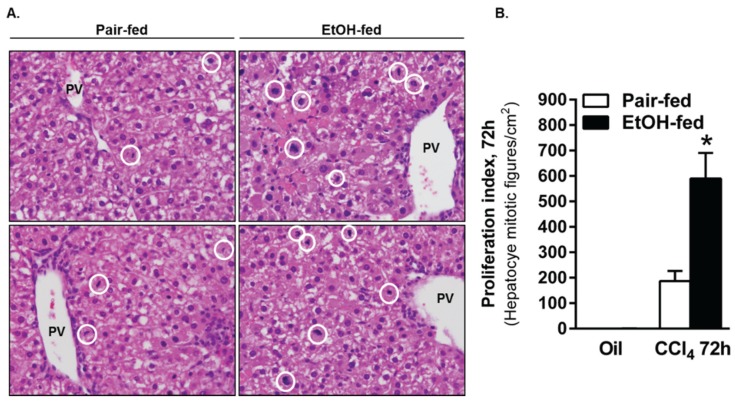
The number of mitotic figures was greater in livers from ethanol-fed mice 72 h after CCl_4_ exposure. (**A**) Representative images from H&E stained liver sections taken from mice 72 h after CCl_4_ exposure. Mitotic figures were found predominantly in Zones 1 and 2, so the images were captured using the portal vein as a landmark. The white circles in each image outline mitotic cells. (**B**) Graphical representation of the number of mitotic cells in livers from pair and ethanol-fed mice 72 h after CCl_4_ exposure. Mitotic figures were rare or not present at other time points evaluated. *N* = 4–8 mice per group. * *p* < 0.05.

Changes in cyclin expression and Rb phosphorylation were followed up by an analysis of hepatocyte proliferation assessed by Ki67 immunostaining and quantification of mitotic figures in H&E-stained liver sections. Using Ki67 immunofluorescence, we observed robust hepatocyte proliferation in livers from pair- and ethanol-fed mice 48 h after CCl_4_ exposure, which was not different between diet groups (Figure 8). However, as the proliferative response waned, 5.5-fold more Ki67-positive cells were found in livers from ethanol-fed mice when compared to pair-fed mice 96 h after CCl_4_. Moreover, the number of mitotic figures was three-fold greater in livers from ethanol-fed mice 72 h after CCl_4_ (Figure 9). It is interesting to note that mitotic figures were rare 96 h after CCl_4_ and not different between pair- and ethanol-fed mice, despite the increased Ki67-positive staining at this time point. This apparent discrepancy suggests either fewer cells in livers from ethanol-fed mice had exited the cell cycle compared to cells in livers from pair-fed mice, or an additional wave of proliferation occurs in livers from ethanol-fed mice >96 h after CCl_4_ exposure. Additional studies would be required to determine the reason for this apparent disconnect.

Collectively, the data in Figure 6, Figure 8 and Figure 9 suggest that moderate ethanol exposure induces a more robust hepatic regenerative response after acute CCl_4_ exposure. While some data suggest enhancement of various indices of the regenerative response at any given time point, other data suggest that the overall response may actually be prolonged. Lack of differences in the peak response would support this idea. Regardless, the more robust regenerative phenotype in livers from ethanol-fed mice is likely due to the need to replace the greater number of hepatocytes lost after acute CCl_4_ exposure (Figure 5). Finally, enhanced pro-regenerative signaling (Figure 7) may drive this apparently prolonged period of liver regeneration. In order to ensure that the liver regenerative response is actually prolonged and not just enhanced at late time points (*i.e*., 72–96 h), we would need to evaluate indices of regeneration greater than 96 h after acute CCl_4_ exposure.

#### 2.3.4. Role of Ethanol on Liver Regeneration

Interestingly, our data on the liver regenerative response after “chemical hepatectomy” were similar to those found after partial (surgical) hepatectomy in a high-dose binge (acute) ethanol exposure model (6 g/kg ethanol given three times before partial hepatectomy) [45]. Ding *et al*. observed enhanced hepatocyte proliferation after partial hepatectomy, which was also independent of liver injury. This finding was attributed to an ethanol-dependent increase in mitochondrial aldehyde dehydrogenase activity preventing mitochondrial oxidative stress and preconditioning the liver to a more rapid proliferative response [45]. Additionally, Apte *et al*. demonstrated that chronic ethanol exposure alone impacts hepatocyte proliferation [46]. Specifically, a significant percentage of hepatocytes are in *S*-phase after one, two, or three weeks of ethanol feeding to rats (5% *v*/*v*), but not after four or five weeks of ethanol feeding [46]. These authors also demonstrated that early in feeding paradigm, ethanol exposure enhanced apoptotic cell death; this likely stimulated the transient compensatory hepatocyte division observed in rats fed ethanol for 1–3 weeks. Taken together, while the apparent mechanisms promoting liver regeneration differ between ethanol exposure models, the end result was similar in these studies: ethanol exposure increases indices of hepatocyte proliferation. These data highlight the liver’s ability to appropriately respond to ethanol-mediated cell death, a property which is lost after long-term ethanol exposure.

In contrast to a beneficial effect of ethanol on liver regeneration as described above, liver regeneration after partial hepatectomy in rats after a single acute ethanol exposure (6 g/kg) did not alter ^3^H-thymidine incorporation when given 4 h before, at the time of or 4 or 8 h after partial hepatectomy [47]. However, when ethanol was administered 12 or 16 h after partial hepatectomy, liver regeneration was inhibited [47]. Likewise, chronic high-dose ethanol (4% or 5% *v*/*v* ethanol for five or six weeks) exposure inhibits liver regeneration. Specifically, ^3^H-thymidine incorporation and mitotic index are reduced after acute [47] or chronic ethanol feeding and is due to inhibition of ornithine decarboxylase activity and downstream polyamine synthesis in rats [48], and induction of redox sensitive cell cycle inhibitors in mice [49]. Collectively, the ethanol dose, pattern of exposure and timing of exposure relative to additional hepatic insult differentially regulate the hepatic proliferative response.

### 2.4. Impact of Moderate Ethanol Feeding on Hepatic Stellate Cell Activation and Extracellular Matrix (ECM) Remodeling after Acute CCl_4_ Exposure

#### 2.4.1. Hepatic Stellate Cell (HSC) Activation

**Figure 10:**
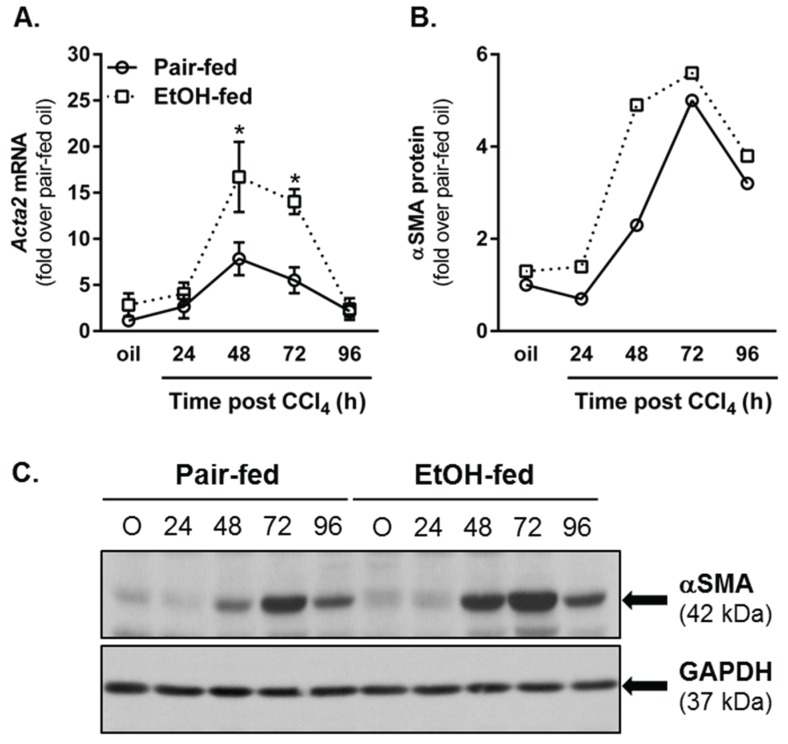

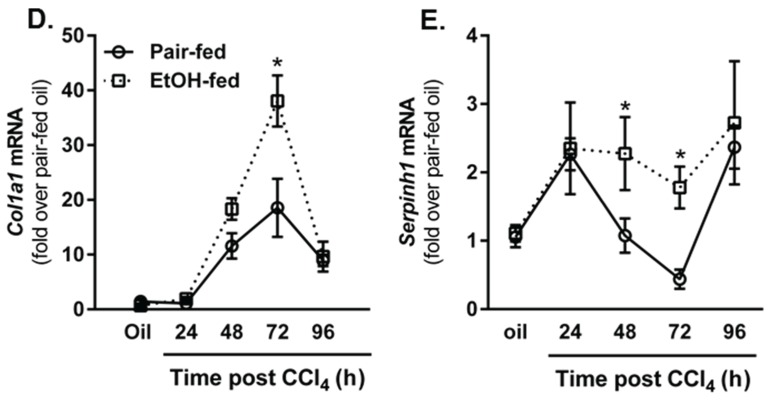
Moderate ethanol feeding enhanced hepatic stellate cell (HSC) activation after acute CCl_4_ exposure. (**A**) Real time PCR was used to measure hepatic *Acta2* transcript accumulation. Data are expressed as fold change over pair-fed, olive oil-exposed mice after normalization to 18S; (**B**) Semi-quantification of αSMA band density expressed as fold change of pair-fed, olive oil exposed mice after normalization to GAPDH as a loading control; (**C**) Representative αSMA immunoblot. Hepatic *Col1a1* (**D**) and *Serpinh1* (**E**) transcript accumulation were determined using real-time PCR. Data are expressed as fold change over pair-fed, olive oil-exposed mice after normalization to 18S. *N* = 4–8 mice per group. * *p* < 0.05.

The later stages of wound healing after tissue injury involve activation of resident fibroblast populations, production of extracellular matrix and matrix remodeling [4]. After liver injury induced by CCl_4_, hepatic stellate cells (HSC) are activated and transdifferentiate into matrix-synthesizing myofibroblasts [50]. HSC upregulate their expression of *Acta2*, the gene which encodes for α smooth muscle actin (αSMA), produce collagen and increase their expression of the collagen specific chaperone, heat shock protein (hsp)47 [50,51]. To determine whether or not moderate ethanol feeding to mice enhanced indices of HSC activation in our hands, we quantified *Acta2* transcript and protein accumulation in livers from pair- and ethanol-fed mice. At 48 and 72 h after CCl_4_ exposure, hepatic αSMA was increased at the mRNA level in livers from both pair- and ethanol-fed mice, but was threefold greater in livers from ethanol-fed mice (Figure 10A). At the protein level, immunoblotting revealed a twofold increase in αSMA protein 48 h after CCl_4_ in livers from ethanol-fed mice; no differences between diet groups were observed at any other time point (Figure 10B,C). Consistently, hepatic transcripts for type I collagen (*Col1a1*) and *Serpinh1* (the gene which encodes the hsp47 protein) were also increased by CCl_4_ exposure in both diet groups, but were increased further in livers harvested from ethanol-fed mice 72 h (*Col1a1*) or 48 and 72 h (*Serpinh1*) after CCl_4_ exposure. Therefore, similar to published work [8,10], CCl_4_-induced HSC activation and induction of markers associated with fibrogenesis were increased by moderate ethanol exposure relative to those increases observed in pair-fed mice.

#### 2.4.2. Matrix Remodeling

**Figure 11:**
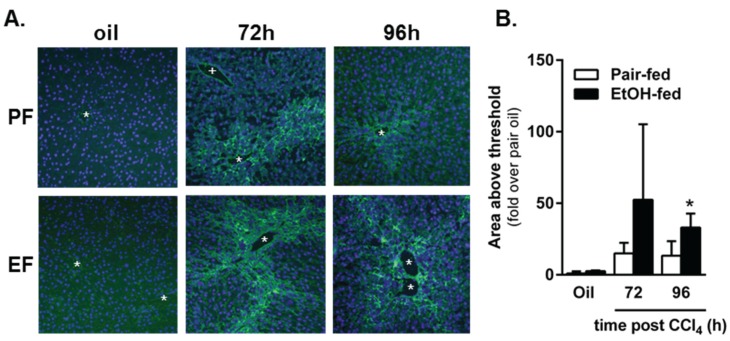
Matrix metabolism was sustained by ethanol feeding to mice after acute CCl_4_ exposure. *In situ* zymography was used to determine area of gelatinase activity in frozen liver sections from pair- and ethanol-fed mice after CCl_4_ exposure. (**A**) Representative fluorescence microscopy images. The green fluorescence indicates area of gelatinase activity. The blue fluorescence (DAPI) was used as a nuclear counter stain. The asterisks denote location of central veins, while the plus sign (PF, 72 h only) denotes the location of a portal vein. Matrix metabolism occurs most robustly in the pericentral area where CCl_4_-induced liver injury and subsequent liver regeneration occurs; (**B**) Quantification of the area of fluorescence per 200× image as a fold change over pair-fed, olive oil-treated mice. PF = Pair-fed, EF = Ethanol-fed. *N* = 3–6 mice per group (CCl_4_). * (in **B**), *p* < 0.05.

In addition to HSC activation and the production of ECM proteins, considerable matrix remodeling also occurs during the liver wound healing response. In the acutely injured liver, ECM degradation facilitates two things: (1) hepatocyte migration to the injured area required for liver regeneration and (2) remodeling of the ECM after liver regeneration is complete. Using *in situ* zymography, we observed matrix degradation 72 and 96 h after acute CCl_4_ exposure in pair- and ethanol-fed mice (Figure 11); there was no matrix degradation at 24 h after CCl_4_, and excessive background staining prevented quantification of matrix degradation at 48 h. While there was a trend toward an increase in matrix degradation in livers from ethanol-fed mice 72 h after CCl_4_, this was not significant. However, 96 h after CCl_4_ exposure, matrix degradation was increased relative to that in pair-fed mice. These data suggest that matrix remodeling is further enhanced by moderate ethanol feeding particularly late in the liver wound healing response. It is likely that this enhanced remodeling period is related to persistent necrotic tissue and prolonged regenerative response found in livers from ethanol-fed, CCl_4_-exposed mice (Figure 3). To determine if the response is prolonged and not just enhanced at 96 h, time points greater than 96 h would need to be evaluated.

## 3. Experimental Section

### 3.1. Reagents

Primary antibodies used include: Cytochrome P450 2E1 (CYP2E1, AbCam, Cambridge, MA, USA, polyclonal antibody), and GAPDH (Cell Signaling, Beverly, MA, USA, clone 14C10), cyclin D1 (Cell Signaling, clone 92g2), cyclin E1 (Cell Signaling, clone HE12), cyclin A2 (Cell Signaling, clone BF683), cyclin B1 (Cell Signaling, clone v152), phospho-retinoblastoma on Ser 780 (phospho-Rb, Cell Signaling, clone D59B7), Ki67 (Abcam, polyclonal antibody), α smooth muscle actin (αSMA, AbCam, polyclonal antibody). Appropriate horseradish peroxidase (HRP)-conjugated secondary antibodies (Abcam) or fluorochrome-conjugated secondary antibodies (Ki67 only, Invitrogen, Grand Island, NY, USA) were used as detection reagents. Olive oil and carbon tetrachloride (CCl_4_) were purchased from Sigma-Aldrich (St. Louis, MO, USA), Buprenex analgesic (buprenorphine HCl) was manufactured by Reckitt Benckiser Healthcare (UK), Ltd., Hull England, UK, and distributed by Reckitt Benckiser Pharmaceuticals, Inc., Richmond, VA, USA.

### 3.2. Animal Care

Animals were treated humanely and in accordance with protocols approved by Case Western Reserve University’s (CASE) and University of Kansas Medical Center’s (KUMC) respective Institutional Animal Care and Use Committees. Both Institutions were AAALAC accredited. Female, wild-type (C57BL/6J) mice (10–12 weeks old) were purchased from Jackson Labs (Bar Harbor, ME, USA) and were allowed to acclimate to their new environments for 48 h, in ventilated cages with a 10/14 hour light/dark cycle with access to standard mouse chow and water *ad libitum*. For experimental procedures, mice were housed in static (CASE) or ventilated (KUMC) cages designed for use with toxic agents as required by each University’s animal care and use program and environmental health and safety offices.

### 3.3. Ethanol Feeding

Mice were housed two per cage and were acclimated to a nutritionally-complete, Lieber-DeCarli liquid diet (Dyets, Bethlehem, PA, USA, cat# 710260) for 2 days, after which half of the mice received a diet containing 1% ethanol for 2 days (2d), then 2% ethanol for the remainder of the experiment. Mice ate an average of 13.1 mL of the 2% ethanol-containing diet per day. Plasma ethanol levels are undetectable when measured 3 h into the dark/feeding period [30]. Control mice were pair-fed an isocaloric diet where ethanol calories were substituted by calories from maltose dextrin for the entire experiment. Pair-fed animals received the volume of diet consumed by their ethanol-fed experimental counterparts on the previous day to ensure equivalent calories were consumed between groups. There was no difference in final body weight between pair and ethanol-fed mice at each time point after CCl_4_ when mice were euthanized (Table 1).

### 3.4. Carbon Tetrachloride Exposure, Tissue Collection and Storage

After receiving 2% ethanol for 2 d, or control liquid diets, mice were given a single intraperitoneal (i.p.) injection of CCl_4_ (1 µL/g body weight) prediluted 1:3 in olive oil using 100 µL Hamilton syringes. Subcutaneous administration of an analgesic (Buprenex) preceded each CCl_4_ injection by 10 min, as done previously [52]. Control mice received analgesic and an olive oil injection and were euthanized 72 h later as done previously (refs). At 24, 48, 72 or 96 h post CCl_4_, mice were anesthetized using a cocktail of ketamine, xylazine and acepromazine. Blood was collected from the inferior vena cava into EDTA and aprotinin-containing tubes and placed on ice. After blood was collected, the diaphragm, superior vena cava and aorta were cut euthanizing the mouse. After euthanasia, a hepatectomy was performed. The liver was divided into several pieces while resting on an ice-cold piece of glass: the small half of the median lobe was cut into 3 pieces and placed into 2 mL tubes with 1.5 mL of RNA later, stored on the bench for 5 min, then at 4 °C for 18 h and then transferred to −20 °C until use. The large half of the median lobe was embedded in Optimal Cutting Temperature medium and frozen on a bed of frozen isopentane and then stored at −80 °C. The largest lobe of the liver (left lobe) was cut into several slices some of which were used for Western blot analysis (snap frozen in liquid nitrogen, stored at −80 °C) or fixed in formalin and later embedded in paraffin for histological and immunohistochemical analysis. The right lobe was snap frozen in liquid nitrogen and then stored at −80 °C for triglyceride quantification. All remaining liver tissue is snap frozen and archived at −80 °C; CYP2E1 activity assays were performed using one of these archived liver pieces. Blood was centrifuged at 10,000× *g* for 3.5 min. Plasma was collected and separated into two aliquots and frozen at −80 °C until use.

The table below contains initial and final body weights, liver weights and liver weight as a percentage of body weight.

### 3.5. CYP2E1 Activity Assay

Liver microsomes were prepared by homogenizing 100–150 mg of frozen liver tissue in 1 mL PBS with a loose fitting dounce homogenizer. After separation and removal of fat, 10 mL of PBS was added and the homogenate was ultracentrifuged at 105,000× *g* for 1 h at 4 °C. The pellet was resuspended in 0.15 M KCl and total protein concentration determined by BCA assay (Life Techologies/Pierce, Grand Island, NY, USA). Thirty micrograms of protein was added to 4 μL of 10 mM *p*-nitrophenol, 10 μL phosphate buffer (4 mL, 1 M K_2_HPO_4_ + 1 mL, 1 M KH_2_PO_4_ pH 7.4) and water was added to 100 μL. Ten microliters of freshly prepared NADPH (10 nM) was then added and the samples were incubated at 37 °C in a water bath for 1 h. Following incubation, 30 μL of 20% trichloroacetic acid was added, samples were vortexed, then centrifuged 10,000× *g* for 10 min. One hundred microliters of supernatant was added to 10 μL of 10 N NaOH and absorbance was determined at 510 nm. CYP2E1 activity was calculated using the extinction coefficient of 9.53 × 10^5^ M^−1^ cm^−1^, normalized to protein concentration and expressed as fold change over wild-type, oil-exposed mice.

### 3.6. Liver Injury and Steatosis Determination

Plasma alanine aminotransferase (ALT) activity was determined using a commercially available enzymatic assay (Sekisui Diagnostics, Exton, PA, USA) according to the manufacturer’s instructions. Activity was calculated using the extinction coefficient method. For triglyceride measurement, livers were digested with 3 M KOH in 65% ethanol for 1 h at 70 °C and vortexed every 20 min. Twenty-four hours later, triglyceride GPO reagent (Pointe Scientific, Canton, MI, USA) and a standard curve created using a GPO standard, were used to calculate total hepatic triglyceride content after absorbance readings at 500 nm were measured.

### 3.7. Histopathologic Analysis

Blinded histological assessment was performed by a board-certified pathologist. Hematoxylin and eosin (H&E)-stained liver sections were examined using a light microscope (Olympus BX-41, Olympus, Waltham, MA, USA); the following characteristics were evaluated: estimated percent necrosis, zone in which necrosis was present, estimated percent steatosis, and the presence, location and severity of inflammation.

### 3.8. RNA Isolation, cDNA Synthesis and Real-Time Polymerase Chain Reaction (PCR)

Total RNA was isolated from RNALater stabilized liver pieces (20–30 mg) using the Qiagen RNeasy Mini Kit (Valencia, CA, USA) after homogenization using the MP Biomedicals Fast Prep 24 bead homogenizer with lysing matrix D homogenization tubes (Solon, OH, USA). Four micrograms of RNA was reverse transcribed into cDNA using the Retroscript kit (Life Technologyies/Ambion, Grand Island, NY, USA). SYBR green (Universal Super Mix, BioRad, Hercules, CA, USA) was used for real-time PCR performed in a BioRad CFX384. Results were calculated using 2^−ΔΔCt^ method. The data were expressed as fold change over pair-fed, olive oil-treated mice. Primers utilized in this study are found in Table 2; 18S was used as the housekeeping gene and did not differ between genotypes or time points after CCl_4_. Sequence sources are noted in the table, most of which were obtained from the PrimerBank [53,54,55].

**Table 2:** Primers used for real-time PCR transcript analysis.

Gene Name	Sequence Source	Forward Primer	Reverse Primer
*Tnf*	[13]	CCCTCACACTCAGATCATCTTCT	GCTACGACGTGGGCTACAG
*Emr1*	PrimerBank: 183583543b1	CTGCACCTGTAAACGAGGCTT	TTGAAAGTTGGTTTGTCCATTGC
*Ly6c*	PrimerBank: 26353880a1	GCAGTGCTACGAGTGCTATGG	ACTGACGGGTCTTTAGTTTCCTT
*Ccnd1*	[52]	CAGAAGTGCGAAGAGGAGGTC	TCATCTTAGAGGCCACGAACAT
*Ccne1*	PrimerBank: 6671698a1	GTGGCTCCGACCTTTCAGTC	CACAGTCTTGTCAATCTTGGCA
*Ccna2*	PrimerBank: 161353443c2	GCCTTCACCATTCATGTGGAT	TTGCTGCGGGTAAAGAGACAG
*Ccnb1*	PrimerBank: 28195398a1	AAGGTGCCTGTGTGTGAACC	GTCAGCCCCATCATCTGCG
*Acta2*	PrimerBank: 31982518b1	CCCAGACATCAGGGAGTAATGG	TCTATCGGATACTTCAGCGTCA
*Col1a1*	[56]	CAAGAACAGCAACGAGTACCG	GTCACTGGTCAACTCCAGCAC
*Serpinh1*	PrimerBank: 6753304a1	GCCGAGGTGAAGAAACCCC	CATCGCCTGATATAGGCTGAAG

### 3.9. TNFα Enzyme-Linked Immunosorbent (ELISA) Assay

TNFα peptide levels were determined from plasma samples collected from pair- and ethanol-fed mice at baseline (oil), 24, 48, 72 and 96 h after CCl_4_ exposure using and ELISA (R&D Systems, Minneapolis, MN, USA) according to the manufacturer’s instructions.

### 3.10. Terminal Deoxynucleotidyl Transferase-Mediate dUTP Nick End Labeling (TUNEL) Assay Procedure, Image Acquisition and Data Collection

Apoptotic hepatic DNA fragmentation was detected by TUNEL using the ApopTag Plus fluorescence *in situ* apoptosis detection kit (Millipore, Temecula, CA, USA) according to manufacturer’s instructions. The fluorescence was quantified as described earlier [52].

### 3.11. Immunoblotting

Liver lysates were prepared as described [52]. Samples were resolved on 10% SDS-PAGE gels after which total protein was transferred to PVDF membranes, blocked in 5% non-fat dry milk and then probed for proteins of interest overnight at 4 °C with agitation. HRP-conjugated secondary antibodies were used, and after an Enhanced Chemiluminescent substrate (GE Healthcare, Piscataway, NJ, USA) was applied to the membranes, luminescence was captured using radiographic film. Quantification of band density was achieved using ImageJ (National Institutes of Health, Bethesda, MD, USA). Data were normalized to a housekeeping gene (GAPDH) and data were expressed as fold change over pair-fed mice exposed to olive oil.

### 3.12. Ki67 Immunofluorescence Assay, Image Acquisition and Data Collection

Frozen sections (5 μm) were cut and fixed with 10% buffered formaldehyde for 10 min at room temperature (RT). All sections were treated with 0.1% Triton X-100 in PBS for 15 min at room temperature, washed three times for 2 min each in PBS, and incubated for a further 1 h with 10% normal donkey serum (Jackson ImmunoResearch Laboratories, Inc., West Grove, PA, USA) as a blocking agent. After incubation, the Ki67 polyclonal antibody solution (1:500 in 1% donkey serum) was applied to tissue sections overnight at 4 °C. The next day, the sections were washed and incubated with a fluorochrome-conjugated donkey-anti-rabbit secondary antibody (1:500, Invitrogen) for 1 h at RT. Nuclei were counterstained with 4,6-diamino-2-phenylindone (DAPI). An Olympus BX51 microscope with an Olympus BH2RFLT3 burner, Olympus DP71 camera and DP Controller software were used to capture 3 non-overlapping images from each tissue section at 200× magnification. The camera settings were chosen to minimize autofluorescence but maintain positive signal. Those settings were used for each image acquired. ImageJ was used to quantify the number of Ki67-positive hepatocyte nuclei (determined based on cell and nuclear morphology) by a blinded individual. Data were expressed as number of Ki67 positive hepatocyte nuclei per 200× image.

### 3.13. Mitotic Figure Quantification

Formalin-fixed, paraffin-embedded sections were cut (5 µM) and stained with hematoxylin and eosin (H&E). Micrographs were taken at 200× magnification using an Olympus BX51 microscope fitted with an Olympus DP71 camera. DP Controller software were used to acquire images (Olympus, Waltham, MA, USA). Four non-overlapping images per liver section were acquired, each of which contained a portal triad, as this is the area where mitotic figures were found. Mitotic index was determined by counting the number of mitotic figures (any mitosis stage) in each image by a blinded individual.

### 3.14. In Situ Zymography, Image Acquisition and Data Collection

Frozen tissue sections (7 μm) were taken from −80 °C and immediately incubated with developing buffer (100 mM Tris, pH 7.4, 100 mM NaCl, 5 mM CaCl_2_, 0.05% Brij-35, 0.25 mM PMSF) containing 0.1 mg/mL Oregon green, dye quenched (DQ) gelatin (Life Technologies/Molecular probes, Grand Island, NY, USA). The slides were incubated in a humid chamber at 37 °C for 16–18 h. After this incubation, a DAPI-containing solution was used as a nuclear counterstain and aqueous mounting medium. An Olympus BX51 microscope with an Olympus BH2RFLT3 burner, Olympus DP71 camera and DP Controller software were used to capture 3 non-overlapping images from each tissue section at 200× magnification. The camera settings were chosen to minimize autofluorescence but not lose positive signal. Those settings were used for each image. ImageJ was used to quantify area of the fluorescent signal generated by matrix metabolism.

### 3.15. Statistics

All results are presented as means ± SEM. Statistical significance was defined as *p* ≤ 0.05 and denoted with *. Students *t*-test was used when comparing two datasets and ANOVA with a Tukey’s adjustment for multiple comparisons was used when comparing time course data.

## 4. Conclusions

**Figure 12:**
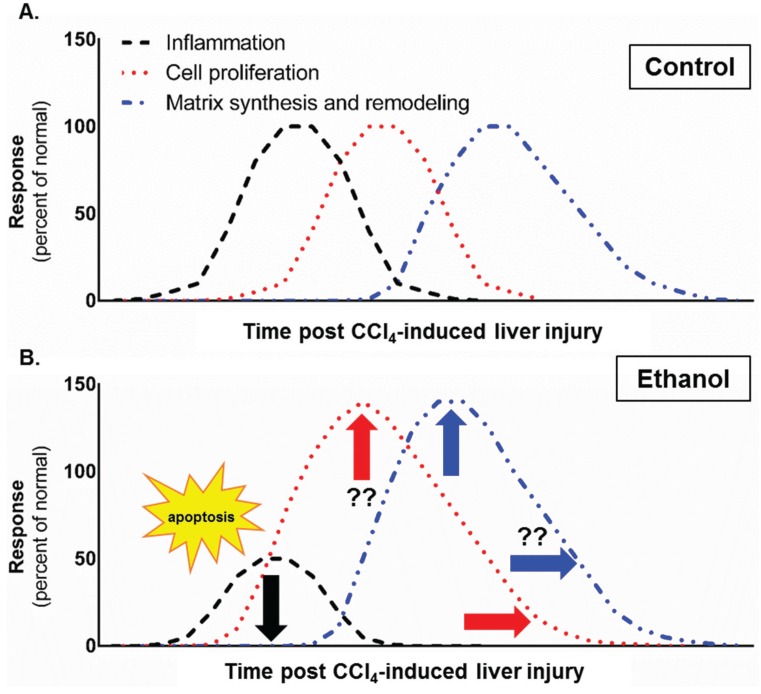
Effect of moderate ethanol feeding to mice on the liver wound healing response after acute hepatotoxin exposure. In (**A**), three phases of wound healing, inflammation (black dotted line), cell proliferation (red dotted line) and HSC activation, matrix synthesis and remodeling (blue dotted line) are depicted as overlapping curves which peak at 100% of normal response after acute CCl_4_; In (**B**), the effect of moderate ethanol on these three phases of wound healing after acute CCl_4_ exposure are depicted. The arrows added to the graph indicate that ethanol decreased (black downward arrow), prolonged (red right arrow) or enhanced (blue upward arrow) the related phase of the wound healing response. In brief, the data contained in this study demonstrated that early after CCl_4_-induced acute liver injury, inflammation is reduced in ethanol-fed mice relative to pair-fed mice. Reduced inflammation was associated with increased hepatocyte apoptosis (yellow “apoptosis” star), which occurred in parallel with a prolonged hepatocyte proliferative response. Moreover, indices of HSC activation were enhanced (peak values greater) in ethanol-fed mice. Finally, matrix degradation was prolonged in livers from ethanol-fed mice. Question marks indicate that more data is required to determine whether or not the ethanol-mediated effect, enhancement or prolongation, occurs in that phase of the wound healing response. We propose that reduction in TNFα, a surrogate marker for hepatoprotective inflammation, in ethanol-fed mice was responsible for increased apoptotic hepatocyte death and, therefore, the need for more robust hepatocyte proliferation, ECM synthesis and remodeling after CCl_4_-induced acute liver injury.

This study evaluated the impact of moderate ethanol feeding on some parameters associated with each phase of the liver wound healing response induced by acute CCl_4_ exposure. Our data suggest that suppression of inflammation early in the wound healing response precipitated expansion of liver injury. Expansion of liver injury was independent of CCl_4_-induced hepatocyte necrosis, but dependent on hepatocyte apoptosis. Given published studies, which demonstrate TNFα protects hepatocytes from apoptosis [11,12,13], we postulate that reduced TNFα in livers from ethanol-fed mice contributed to increased hepatocyte apoptosis. Perhaps as a compensatory response to the increased hepatocyte loss, the proliferative and matrix remodeling phases of the hepatic wound healing response in ethanol-fed mice were prolonged relative to that found in pair-fed mice after CCl_4_ exposure (Figure 12). These data help fill some gaps in our knowledge of how moderate ethanol affects wound healing after liver injury. In addition, our study suggests that moderate ethanol (two drinks per day), though considered beneficial in otherwise healthy individuals [57,58,59], can accelerate liver damage and fibrogenic changes in the liver after exposure to hepatotoxic agents, *i.e*., in industrial or agricultural settings. While this may appear beneficial in the context of acute liver injury as an appropriate response, long term exposure to hepatotoxic agents in individuals who regularly consume moderate amounts of ethanol may be more likely to progress to fibrosis, cirrhosis or hepatocellular carcinoma than those who abstain from drinking alcohol.
